# Correct positioning of the calcar screw leads to superior results in proximal humerus fractures treated with carbon-fibre-reinforced polyetheretherketone plate osteosynthesis with polyaxial locking screws

**DOI:** 10.1186/s10195-023-00733-3

**Published:** 2023-10-10

**Authors:** Michael Kimmeyer, Jonas Schmalzl, Verena Rentschler, Christian Schieffer, Arno Macken, Christian Gerhardt, Lars-Johannes Lehmann

**Affiliations:** 1 Department of Traumatology, Hand Surgery and Sports Medicine, ViDia Clinics Karlsruhe, Steinhaeusserstr. 18, 76135 Karlsruhe, Germany; 2 Department of Trauma, Hand, Plastic and Reconstructive Surgery, University Hospital Wuerzburg, Oberduerrbacher Str. 6, 97080 Wuerzburg, Germany; 3 Department of Orthopaedics and Sports Medicine, Erasmus Medical Centre, Dr. Molewaterplein 40, 3015 GD Rotterdam, The Netherlands; 4 Alps Surgery Institute, Clinque Générale d’Annecy, 4 Chemin de la Tour la Reine, 74000 Annecy, France

**Keywords:** Angular stable plate osteosynthesis, PEEK, Avascular necrosis, Varus dislocation, Subjective shoulder value, Constant–Murley score

## Abstract

**Background:**

Plate osteosynthesis with implants made of carbon-fibre-reinforced polyetheretherketone (CFR-PEEK) has recently been introduced for the treatment of fractures of the proximal humerus (PHFs). The advantages of the CFR-PEEK plate are considered to be its radiolucency, its favourable modulus of elasticity, and the polyaxial placement of the screws with high variability of the angle. The primary aim of this study is to investigate the influence of calcar screw positioning on the complication and revision rates after CFR-PEEK plating of PHFs. The secondary aim is to assess its influence on functional outcome.

**Material and methods:**

Patients were identified retrospectively. Minimum follow-up was 12 months. The cohort was divided into two groups depending on the distance of the calcar screw to the calcar (group I: < 12 mm, group II: ≥ 12 mm). The range of motion (ROM), Subjective Shoulder Value Score (SSV) and Constant–Murley Score (CS) were analysed at follow-up examination. Subjective complaints, complications (e.g. humeral head necrosis, varus dislocation) and the revision rate were evaluated.

**Results:**

51 patients (33 female, 18 male) with an average age of 68.6 years were included after a period of 26.6 months (group I: 32 patients, group II: 19 patients). Apart from the gender distribution, no significant differences were seen in the patient characteristics. The outcome scores showed significantly better clinical results in group I: SSV 83.4 vs 71.2, *p* = 0.007; CS 79.1 vs 67.8, *p* = 0.013. Complications were seen less frequently in group I (18.8 % vs 47.4 %, *p* = 0.030).

**Conclusion:**

This study shows that the positioning of the calcar screw is relevant for CFR-PEEK plate osteosynthesis in PHFs with a good reduction of the fracture. Optimal positioning of the calcar screw close to the calcar (< 12 mm) is associated with a lower rate of complications, resulting in significantly superior functional outcomes.

*Level of evidence*: III, retrospective cohort study

## Background

Approximately 5% of all fractures in humans are proximal humerus fractures (PHFs). PHFs are more common in elderly patients, women and patients with a reduced bone density [5]. The treatment of PHFs depends on the fracture morphology, bone quality and patient-specific criteria such as age, physical activity or comorbidities. PHFs are often treated with angular stable plate fixation [13]. Carbon-fibre-reinforced polyetheretherketone (CFR-PEEK) plates have recently been introduced as an alternative to the most commonly used titanium plates [11, 19, 23, 30]. There are several biomechanical advantages of CFR-PEEK plates, such as higher stability of the locking screws, a favourable modulus of elasticity and no cold welding [10, 24]. There are also intraoperative advantages, such as the radiolucency of the plates and the possibility of polyaxial screw placement [10]. In most of the conventional plates, the angle of the drilling of the screws is determined by the plate design [20]. It can be placed under visual control using an image intensifier, depending on the surgeon's preferences, and optimal placement of the screw can then be achieved. In a complex fracture with a comminuted calcar region, the medial stability should be supported to avoid a secondary varus dislocation [17]. There are several technical options, like calcar screws, bone graft augmentation, cement augmentation, additional free screws or double plating [6, 12, 28]. The positioning of a calcar screw has proven useful for inferomedial support to reduce the secondary loss of reduction [14, 16]. Accurate positioning of the calcar screw is important; it should be in the medial quarter of the proximal humerus near the calcar region [1]. Care should also be taken to ensure that the calcar screw is of sufficient length that the screw tip is placed subchondrally [15, 18].

To our knowledge, no previous study has analysed the positioning of polyaxial calcar screws in CFR-PEEK plates in PHFs. The primary aim of this study is to investigate the influence of calcar screw positioning on the complication and revision rates. The secondary aim is to assess its influence on the functional outcome.

## Material and methods

After approval from the institutional research ethical committee, patients were retrospectively identified for this study from the electronic patient records in a regional trauma center which is also a certified center of shoulder surgery.

### Study population

All procedures between January 2017 and October 2020 adhering to the following inclusion criteria were identified:

- A four-fragment PHF treated with a CFR-PEEKPower^TM^ Humeral Fracture Plate (PEEKPower^TM^ Humeral Fracture Plate (HFP), Arthrex^®^, Naples, United States of America)
- Performed by a single surgeon (L.L.)
- Isolated fracture of the proximal humerus
- Satisfactory reduction and refixation of the fracture in postoperative radiographs
- Surgery was performed within 14 days after trauma
- Minimum follow-up of 1 year
- A signed consent form.

Patients who could not attend the follow-up examination for medical reasons, who did not want a follow-up examination for personal reasons and who could not be contacted were excluded. A declaration of informed consent was signed at the follow-up examination. The data were only collected after a signed declaration of consent.

### Surgical treatment

The specific treatment decision was based on fracture morphology, bone quality and patient-specific criteria (e.g. age, physical activity, comorbidities). The risk of osteosynthesis failure of the proximal humerus was estimated following the criteria defined by Hertel [9]. If the surgeon considered the risk of complications to be high, a primary endoprosthetic joint replacement was performed instead of an osteosynthesis. In patients with preoperative symptomatic osteoarthritis, implantation of a reverse total shoulder arthroplasty (RSA) was preferred. None of the cases were intraoperatively converted to endoprosthetic replacement. The surgery was performed in the beach-chair position under general and/or regional anaesthesia using an interscalene plexus block. A deltopectoral approach was used in all cases. A three- or five-hole CFR-PEEK plate (PEEKPower^TM^ Humeral Fracture Plate (HFP), Arthrex^®^) was used. A titanium calcar screw (soft bone locking screw, 4-mm Arthrex^®^) was placed as close as possible to the calcar. The CFR-PEEK plate allows an angular deviation of the locking screws of up to 12° in all directions. The lengths of the humeral head screws were selected so that their tips extended to the subchondral surface of the humeral head without penetration of the articular surface. Depending on the fracture morphology, additional screws for the lesser tuberosity and a suture cerclage of the tuberosities (FiberWire^®^, Arthrex^®^) were applied, and a tenotomy or tenodesis of the long head of the biceps tendon (LBT) was performed. Depending on the fracture, the follow-up treatment included early functional therapy or a restrictive protocol of immobilization in a shoulder abduction splint for 3 weeks with subsequent passive mobilization of the shoulder. Active rehabilitation of the operated shoulder was started after 6 weeks.

### Evaluation of the functional results and revision surgery

General information (gender, dominant hand, diabetes, current smoking, height, weight) was gathered. Active and passive range of motion of the shoulder (abduction, flexion, external rotation, internal rotation) were assessed by the senior author (M.K.) at the follow-up examination after at least 12 months postoperatively and the Constant–Murley Score (CS) was completed. Isometric force measurement to determine the CS was performed with the patient seated with the shoulder in 90° abduction, 0° anteversion, and the elbow in extension [29]. The strap of the electrical force measurement device was applied to the distal forearm (IsoForceControl® EVO2, Herkules Kunststoff AG, Oberburg, Switzerland). Patient-reported outcome measures (PROMs) were collected, included a Visual Analog Scale for pain (VAS), the Subjective Shoulder Value (SSV) and the Quick Disabilities of the Arm, Shoulder and Hand Score (QDASH). Complications such as adhesive capsulitis, implant loosening or breakage, refracture, secondary fracture dislocation, avascular necrosis of the humeral head, secondary osteoarthritis, mal-/non-union, hematoma and iatrogenic nerve lesions as well as revision surgery (indication, performed surgery) were recorded.

### Radiological analysis

Preoperative radiographs (true anterior posterior (AP) view, lateral (Y) view) and computed tomography (CT) scans were analysed. Both radiographs were also evaluated 2 days after surgery and at the final follow-up. The PHFs were classified according to Codman’s four-fragment theory [4] by analysing CT scans (M.K., V.R.). Furthermore, the presence of a head split component and preexisting glenohumeral osteoarthritis (classified according to Samilson-Prieto [22]) was analysed.

**Fig. 1:**
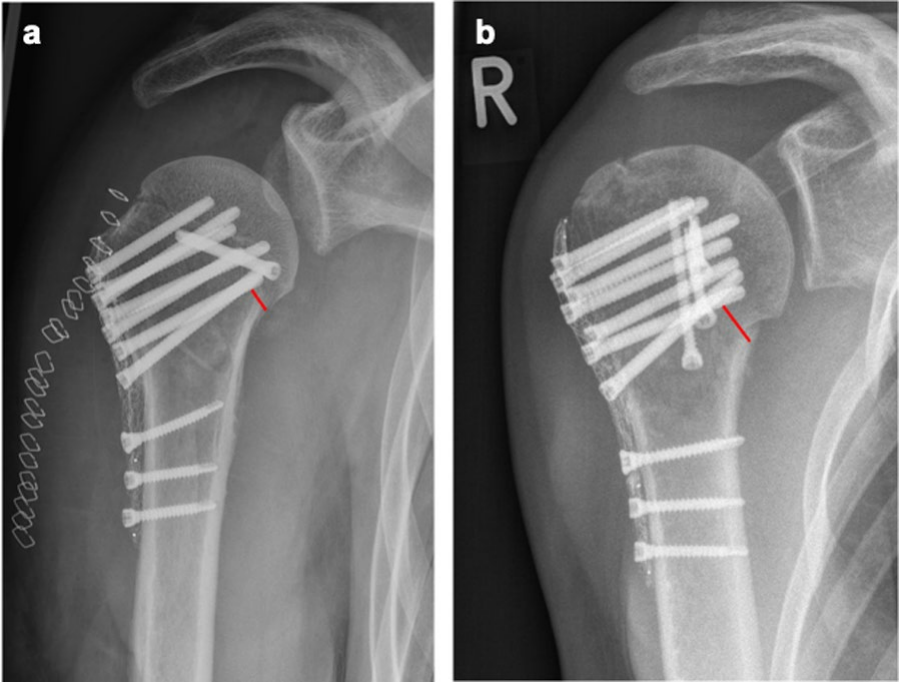
Radiological measurement of the distance from the calcar screw to the calcar in an anterior posterior radiograph: **a** calcar screw distance < 12 mm (group I), **b** calcar screw distance ≥ 12 mm (group II)

**Fig. 2:**
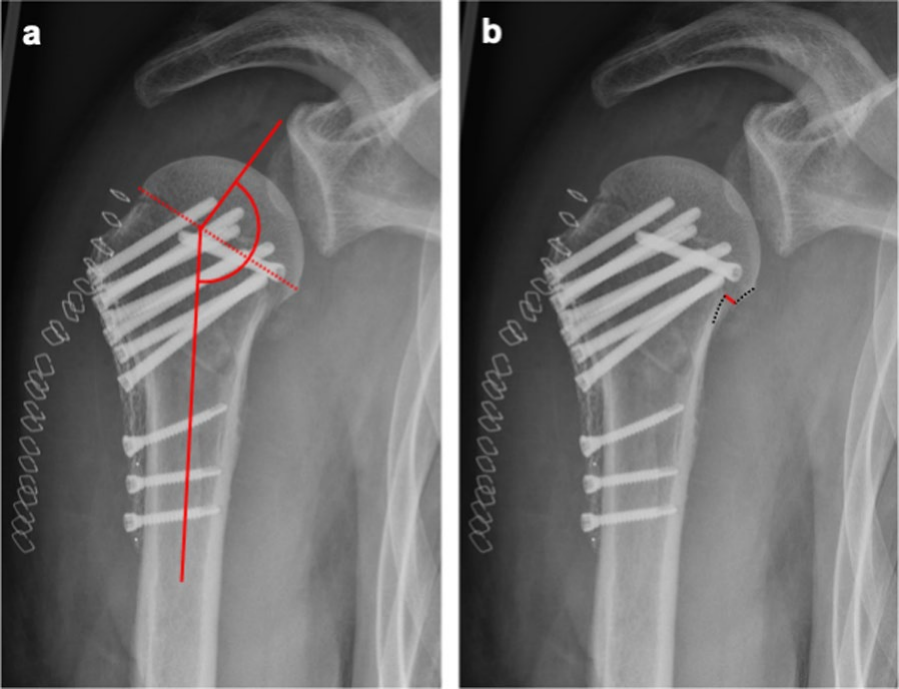
Radiological measurements in an anterior posterior radiograph: **a** neck shaft angulation, **b** neck shaft distance

To evaluate the positioning of the calcar screw, the distance between the calcar and calcar screw (Fig. 1) was measured in postoperatively performed AP radiographs. Fracture reduction was assessed on AP radiographs by measuring the neck shaft angulation (NSA) (Fig. 2a), neck shaft distance (Fig. 2b) and reduction of the greater tuberosity. A satisfactory NSA was defined as being between 110° and 150° [26]. The neck shaft distance (NSD) was measured to quantify the reduction at the medial hinge of the proximal humerus. A satisfactory reduction was defined at a distance of less than 5 mm.

At the final follow-up examination, the following radiographic parameters were assessed: screw or plate breakage or dislocation, non-union, and osteonecrosis of the humeral head. In addition, the integrity and position of the tuberosities (resorption, dislocation ≥ 5 mm) were assessed. Secondary varus dislocation was defined as an angular deviation of more than 110° in AP radiographs. The radiological images were assessed by two orthopaedic surgeons (M.K., V.R.). In the case of disagreement, the final assessment was determined through discussion and consensus.

### Statistical analysis

Two groups were created based on the calcar screw placement: group I: < 12 mm, group II: ≥ 12 mm [18]. Statistical analyses were performed using SPSS^®^ software (version 28.0; IBM^®^, Armonk, United States of America). The nominal variables were summarized as percentages. The arithmetic mean and its standard deviation were used for descriptive statistics in the case of a normal distribution. The Shapiro–Wilk test was used to test the normality of the variables. The Wilcoxon–Mann–Whitney test (*U* test) was used for normally distributed quantitative variables. Pearson’s chi-squared test was calculated to test the association of two ordinal variables. The level of significance was set at *p* < 0.05.

## Results

A total of 79 patients underwent surgery for four-fragment PHF. Four patients were excluded due to nonanatomic repositioning and postoperative malposition of the tuberosities. Of the 75 patients, 24 were unavailable for follow-up, resulting in a loss to follow-up of 32%.

A total of 51 patients (33 females, 18 males; mean age 68.6 ± 11.3 years, range: 45–93 years) were included after a mean follow-up time of 26.6 ± 11.9 (range: 12–58) months. The study collective contained 32 patients in group I and 19 patients in group II. The mean distance of the calcar screw from the medial edge of the calcar was 7.7 ± 2.8 mm in group I and 14.9 ± 2.1 mm in group II (Table 1). Between the two groups, there were no significant differences in terms of age, follow-up time, body mass index, smoking, and diabetes. However, the gender distribution showed a significant difference (group I was 88% females and group II was 53% females, *p* = 0.006). Also, there were no significant differences between the two groups in preoperative radiological parameters such as head-split component or asymptomatic osteoarthritis (Table 2). Furthermore, there were no significant differences in postoperative radiological parameters such as NSD and NSA (Table 3).

**Table 1:** Study population

	Group I (< 12 mm), *n* = 32	Group II (≥ 12 mm), *n* = 19	*p* value
Follow-up (months)	**26.6** ± 12.0	**26.6** ± 12.1	No sign.*
Age (years)	**67.6** ± 12.4	**70.3** ± 9.1	No sign.*
Gender			
Female	**88%** (*n* = 28)	**53%** (*n* = 10)	*p* = 0.006**
Male	**13%** (*n *= 4)	**47%** (*n* = 9)	
BMI (kg/m^2^)	**24.7** ± 3.6	**27.0** ± 4.3	No sign.*
Smoking	**6%** (*n *= 2)	**11%** (*n* = 9)	No sign.**
Diabetes type II	**13%** (*n *= 4)	**0%** (*n* = 0)	No sign.**
Calcar screw distance (mm)	**7.7** ± 2.8	**14.9** ± 2.1	*p* < 0.001*
Plate			
3-Hole	**97%** (*n* = 31)	**89%** (*n* = 17)	No sign.**
5-Hole	**3%** (*n* = 1)	**11%** (*n* = 2)	
Extra screw for lesser tuberosity	**34%** (*n* = 11)	**37%** (*n* = 7)	No sign.**
Therapy for the LBT			
Tenodesis	**6%** (*n* = 2)	**21%** (*n* = 4)	No sign**
Tenotomy	**22%** (*n *= 7)	**16%** (*n* = 3)	
Postoperative treatment			
Functional	**69%** (*n* = 22	**58%** (*n* = 11)	No sign.**
Restrictive	**31%** (*n* = 10)	**42%** (*n* = 8)	

Mean values, percentaged and significant *p* values were highlighted in bold

*n* number, *BMI* body mass index, *LBT* long head of the biceps tendon, *No sign.* No significant difference, *Wilcoxon–Mann–Whitney test, **Pearson’s chi-squared test, *p* < 0.05

**Table 2:** Preoperative radiological outcome

	Group I (< 12 mm), *n* = 32	Group II (≥ 12 mm), *n* = 19	*p* value
Asymptomatic arthrosis signs	**28%** (*n* = 9)	**32%** (*n* = 6)	No sign.**
Varus dislocation < 120°	**23%** (*n* = 7)	**20%** (*n* = 5)	No sign.**
Head split component	**47%** (*n* = 15)	**58%** (*n* = 11)	No sign.**
Neck shaft dislocation (mm)	**6.0** ± 4.1	**6.6** ± 2.8	No sign.*
Metaphyseal head extension (mm)	**8.8** ± 6.1	**7.3** ± 5.6	No sign.*
Tuberosity dislocation (mm)	**10.2** ± 4.1	**12.1** ± 2.8	No sign.*

*n* number

*Wilcoxon–Mann–Whitney test, **Pearson’s chi-squared test, *p* < 0.05

**Table 3:** Postoperative radiological outcome

	Group I (< 12 mm), *n * = 32	Group II (≥ 12 mm), *n *= 19	*p* value
NSD (mm)	**2.3** ± 1.9	**3.8** ± 3.1	No sign.*
NSA (°)	**130.6** ± 9.8	**128.1** ± 9.3	No sign.*

*n* number, *NSA* neck shaft angulation, *NSD* neck shaft distance

*Wilcoxon–Mann–Whitney test, *p* < 0.05

**Fig. 3:**
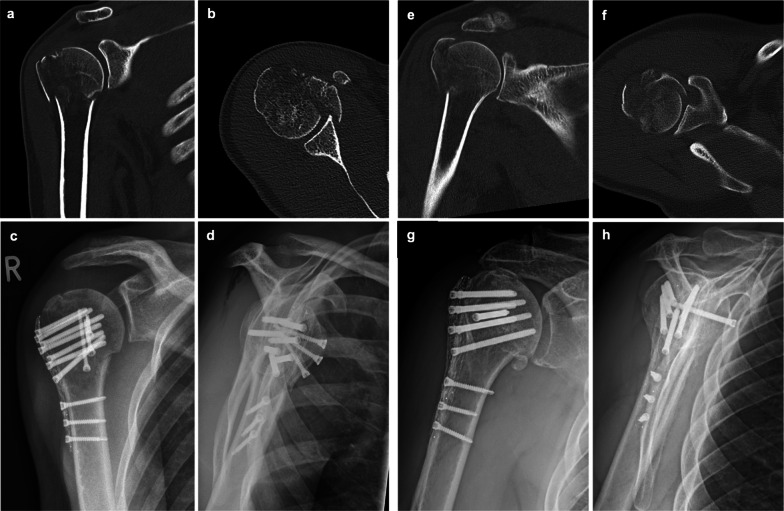
51-year-old female patient with four-part proximal humerus fracture and head split component treated with a CFR-PEEK plate osteosynthesis and additional screws for the lesser tuberosity: **a** preoperative CT scan in a coronal plane; **b** preoperative CT scan in an axial plane; **c** postoperative radiograph in AP view; **d** postoperative radiograph in lateral view; 58-year-old male patient with four-part proximal humerus fracture treated with a CFR-PEEK plate osteosynthesis and an additional screw for the lesser tuberosity;** e** preoperative CT scan in a coronal plane, **f** preoperative CT scan in an axial plane; **g** postoperative radiograph in AP view; **h** postoperative radiograph in lateral view. *CFR-PEEK* carbon-fibre-reinforced polyetheretherketone, *CT* computed tomography

The functional results are shown in Table 4. SSV (83.4 ± 16.3 vs 71.2 ± 15.1, *p* = 0.007), CS (79.1 ± 16.1 vs 67.8 ± 19.3, *p* = 0.013) as well as the QDASH (13.2 ± 16.0 vs 23.4 ±18.4, *p* = 0.026) showed significantly better functional results in group I. Regarding ROM, superior results were seen in group I compared to group II: flexion 147° vs 113°, *p* = 0.002; abduction 146° vs 113°, *p* = 0.007; and external rotation 51° vs 31°, *p* = 0.002 (Fig. 3).

**Table 4:** Functional outcome at follow-up

	Group I (< 12 mm), *N *= 32	Group II (≥ 12 mm), *n* = 19	*p* value
SSV (%)	**83.4** ± 16.3	**71.2** ± 16.1	***p*** = 0.007^*^
CS	**79.1** ± 16.1	**67.8** ± 19.3	***p*** = 0.013^*^
QDASH	**13.2** ± 16.0	**23.4** ± 18.4	***p*** = 0.026^*^
VAS pain	**1.3** ± 1.9	**2.1** ± 2.6	No sign.*
Flexion (°)	**146** ± 29	**113** ± 43	***p*** = 0.002^*^
Abduction (°)	**146** ± 34	**113** ± 43	***p*** = 0.007^*^
External rotation (°)	**51** ± 19	**31** ± 22	***p*** = 0.002^*^

*n* number, *SSV* Subjective Shoulder Value, *CS* Constant–Murley Score, *QDASH* Quick Disabilities of the Arm, Shoulder and Hand Score, *VAS* Visual Analogue Scale of Pain

*Wilcoxon–Mann–Whitney test, *p* < 0.05

Adverse events occurred in 15 patients (29.4%). There were six (18.8%) adverse events in group I and nine (47.4%) adverse events in group II (*p* = 0.030). AVN (12.5% vs 26.3%, *p *= 0.211) and secondary varus dislocation of more than 10° (18.8% vs 26.3%, *p *= 0.525) were observed less frequently in group I without proven statistical significance.

In a total of six (11.8%) patients, revision surgery was required (group I: one shoulder arthrolysis with implant removal, two reverse arthroplasties; group II: one shoulder arthrolysis with implant removal, one reverse arthroplasty).

Figure 3 shows the radiological images of a case with good calcar screw positioning (Fig. 3a–d) and a case with nonoptimal calcar screw positioning (Fig. 3e–h). The first case (Fig. 3a–d) shows a good radiological result. In the second case (Fig. 3e–g), there is a secondary varus dislocation and an AVN without intra-articular screw penetration.

## Discussion

This retrospective case series included 51 patients with PHFs treated with CFR-PEEK plate osteosynthesis by a single surgeon. Postoperative results were analysed in relation to the positioning of the calcar screw. Based on a radiological analysis by Padegimas et al. in which 168 patients were analysed after PHF and conventional locking plate osteosynthesis, the correct placement of the calcar screws was determined to be less than 12 mm to the calcar [18]. They summarized that a calcar screw that is less than 12 mm from the calcar and in the medial quarter of the humeral head can reduce fixation failure in titanium plate osteosynthesis. In comparison to that study, we investigated not only radiographic but also functional parameters related to the positioning of the calcar screw with polyaxial CFR-PEEK plates. In total, the functional results of our study are comparable to those reported in the literature [3]. In a systematic review by Brorson et al., the included studies showed CSs ranging from 60 to 88 [3]. Compared to those studies, the CS in our collective was 75. After dividing up the collective according to the calcar screw positioning, the CS was found to be significantly higher in patients with optimal calcar screw placement. Therefore, we can conclude that correct placement of the calcar screw less than 12 mm from the calcar is associated with better functional results. Since the analysis showed a significantly lower complication rate and a tendency towards a lower revision rate in group 1, we hypothesize that the better functional outcomes in group I are due to the lower incidence of complications and revision surgeries. Although the complication rate in group II was relatively high, the overall rates of complications (29%) and revisions (12%) in our collective were comparable to the data in the literature. A review of 14 studies of displaced four-part PHFs treated with locking plates (ten case series, three retrospective observational comparative studies, one prospective observational comparative study) showed complication rates of 16 to 64% and reoperation rates of 11–27% [2].

In PHFs, certain complications are observed more frequently. AVN is an important post-traumatic complication occurring in up to 34% of cases after locking plate osteosynthesis of the proximal humerus [2, 7–9, 25, 27]. In our study, AVNs were seen in 13% of group I and 26% of group II. Although the difference between the groups was not significant, the group with calcar screw placement close to the calcar showed a trend towards a lower risk of AVN. We hypothesize that increased medial stability with optimal placement of the calcar screw will improve blood flow to the humeral head and reduce the risk of AVN.

Another important complication is a varus deformity of the humeral head, which might result in a varus collapse. If the medial column is comminuted, the stability of the osteosynthesis is significantly reduced and the risk of a varus deformity of the humeral head is increased [21]. In a cadaveric study by Bai et al. it was found that, with good alignment of the humerus, the calcar screw provides additional stability even if the calcar region is destroyed [1]. In our analysis, a lower incidence of secondary varus dislocation was shown in group I, although a significant difference could not be demonstrated. The trend seen in our analysis has also been demonstrated in various cadaver studies [1, 15].

The calcar screw has proven to be a good biomechanical support. But the biomechanical analysis of Bai et al. also showed that the calcar screw has no biomechanical advantages in the presence of varus deformity of the proximal humerus and if the fracture reduction was insufficient [1]. Therefore, when treating PHFs using CFR-PEEK plate osteosynthesis, attention should not only be paid to adequate reduction and good plate positioning but also to the optimal position of the polyaxial calcar screw. When these essential criteria for adequate fracture fixation are met, good postoperative outcomes can be achieved and the risk of complications and revision surgery can be reduced and patient care improved.

## Limitations

Firstly, the retrospective design of the study is a potential source of bias. The analysis also revealed differences in the surgical technique (tenodesis vs tenotomy of the long head of the biceps tendon, refixation of the tuberosity) and postoperative treatment (early mobilization vs a restrictive protocol). These differences may significantly affect both functional outcome and the occurrence of adverse events. The bias could be eliminated by using a prospective study design or stricter exclusion criteria. However, since this would make the study groups very small, a representative analysis with statistical significance would not have been possible. In addition, the follow-up period was at least 1 year. Most adverse events such as AVNs or implant-associated complications occur within the first postoperative year, but adverse events may also potentially occur outside the study period. These events would not have been captured in the study. In addition, only a small number of patients were included in this study, so it is possible that more significant findings could have been found using a larger cohort. The strength of this study is the representative, homogeneous study population of patients with a four-fragment PHF treated with a CFR-PEEK plate. In addition, to our knowledge, this is the only study to analyse the radiologic and functional effects of polyaxial calcar screw placement in CFR-PEEK plates.

## Summary

CFR-PEEK plate osteosynthesis allows polyaxial placement of the locking screws. An optimal calcar screw placement of less than 12 mm from the calcar results in a significantly lower rate of complications, leading to significantly better functional outcomes. Therefore, in clinical practice, optimal calcar screw placement should be considered for sufficient fracture stabilization.

## Data Availability

Patient consent does not include the publication of the data, so publication of the raw data is not possible. The datasets generated and/or analysed during the current study are not publicly available due to a lack of written consent from the patients, but they are available from the corresponding author on reasonable request.
